# Case Report: Clinical analysis of a cluster outbreak of *chlamydia psittaci* pneumonia

**DOI:** 10.3389/fcimb.2023.1214297

**Published:** 2023-08-10

**Authors:** Yinxia Wu, Xuemei Xu, Yun Liu, Xiangwei Jiang, Hongjing Wu, Jie Yang, Limei He

**Affiliations:** ^1^ Department of Infectious Diseases, Lishui Municipal Central Hospital, Lishui, Zhejiang, China; ^2^ Department of Nephrology Diseases, Lishui Municipal Central Hospital, Lishui, Zhejiang, China

**Keywords:** psittacosis, pneumonia, clustered onset, MNGs, clinical characteristics

## Abstract

**Objective:**

To explore the clinical characteristics and prognosis of clustered cases of psittacosis pneumonia.

**Method:**

We retrospectively analyzed the clinical data of a cluster outbreak of psittacosis pneumonia. The analysis included epidemiological data, clinical symptoms, laboratory results, and prognosis. The diagnosis was made using mNGS and nested PCR technology.

**Result:**

Of the four cases, two had direct contact with diseased poultry while the other two did not. All cases presented with more than 39.5 °C fever and chills. Additionally, significant increases in C-reactive protein, ferritin, creatine kinase, and lactate dehydrogenase were observed in all cases, while absolute lymphocyte count decreased. Case 2 also had increased calcitonin levels. Acute respiratory failure occurred during the treatment of case 1 and case 2, leading to tracheal intubation and ventilator-assisted ventilation. Unfortunately, case 2 passed away due to sepsis and multiple organ dysfunction, while the other cases had a positive prognosis.

**Conclusion:**

mNGS facilitated the early diagnosis of psittacosis pneumonia. It is important to note that there is still a substantial risk of human-to-human transmission in psittacosis pneumonia. Absolute lymphocyte count and calcitonin levels can predict the severity and prognosis of the disease.

## Introduction

Chlamydia psittaci is a gram-negative intracellular parasite only found in poultry and known to cause zoonosis (Knittler and Saches, 2015). Parrot fever pneumonia is primarily transmitted through contact with infected birds or their excretions, with the most common cases being associated with parrots and poultry (Balsamo et al., 2017). The use of mNGS technology increased the reported cases of psittacosis pneumonia in recent years, but most of these cases were solitary instances (Teng et al., 2021), and clustered outbreaks were rarely reported. This article reports 4 cases of a clustered outbreak of *chlamydia psittaci* pneumonia. The cases include 3 members of a single family and another individual who sold ducks to the family. The details of these cases are discussed below.

## Basic information about the cases

This study included four cases, consisting of two males and two females aged 42 to 70 years. Case 1 had a history of hypertension and received long-term regular antihypertensive medications, while the other cases had no underlying diseases. None of them had immune system diseases. The onset dates for the cases were August 18th, August 18th, August 27th, and September 4th, 2021, respectively. Hospital stays occurred on August 21, August 28, August 30, and September 5, 2021.

## Epidemiological data

Case 1 was a poultry seller who sold ducks to case 2. Case 2, case 3, and case 4 were a family of three (**Table 1**).

**Table 1:** Baseline characteristics and clinical manifestations of patients with Chlamydia psittaci pneumonia.

Case	1	2	3	4
**Sex**	Female	Male	Female	Male
**Age**	70	64	63	42
**Occupation**	Poultry salesperson	Farmer	Farmer	Worker
**Past history**	Hypertension	None	None	None
**Contact history**	Poultry (chicken and Ducks)	Purchased ducks from Case 1	Wife of Case 2	Son of Case 2
**Time of onset**	2021/8/18	2021/8/18	2021/8/27	2021/9/4
**Admission time**	2021/8/21	2021/8/28	2021/8/30	2021/9/5
**Temperature Peak (°C)**	39.9	39.8	39.5	39.6
**Initial** **symptoms**	Fever Cough	Fever	Fever	Fever
**Concomitant symptoms**	Chilly sensations blood-tinged sputurm chest tightness Shortness of breath	Cough blood-tinged sputurm chest tightness shortness of breath	Chilly sensations; Feeble; cough	Chilly sensations; dry cough

## Clinical manifestation

All four cases presented with fever and chills, with the highest body temperature between 39.5 °C and 39.9 °C. Cases 1 and 2 experienced chest tightness and shortness of breath and developed acute respiratory failure on the third day (August 23) and the first day (August 28) after admission, respectively. Both cases underwent tracheal intubation and ventilator-assisted ventilation. Case 3 presented with chills, fatigue, and cough. Case 4 also presented with cough (**Table 1**).

## Physical examination

Four cases came to the hospital with clear consciousness. Cases 1 and 2 received sedatives and analgesics after tracheal intubation, making their consciousness unmeasurable. Case 1 exhibited low breathing sounds in the right lung, coarse breathing sounds in the left lung, and audible moist rales in the left lung. Case 2 had coarse breathing sounds and moist rales in both lungs. Cases 3 and 4 had thick respiratory sounds in both lungs, with moist rales audible in the right lung. Physical examinations of the heart, abdomen, and nervous system in all four cases revealed no abnormalities.

## Laboratory and imaging examinations

**Figure 1:**
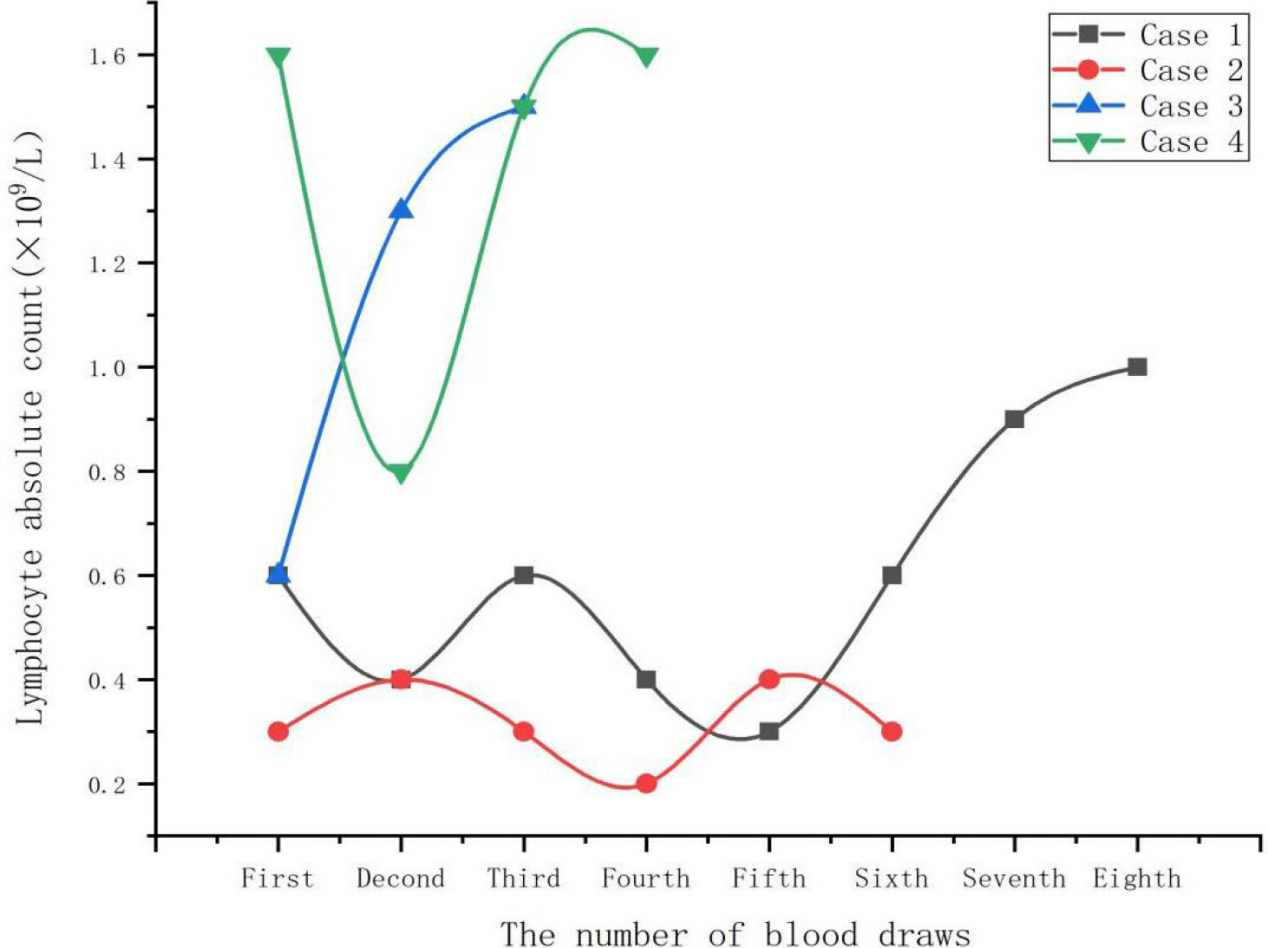
Lympocyte absolute count change curve.

**Figure 2:**
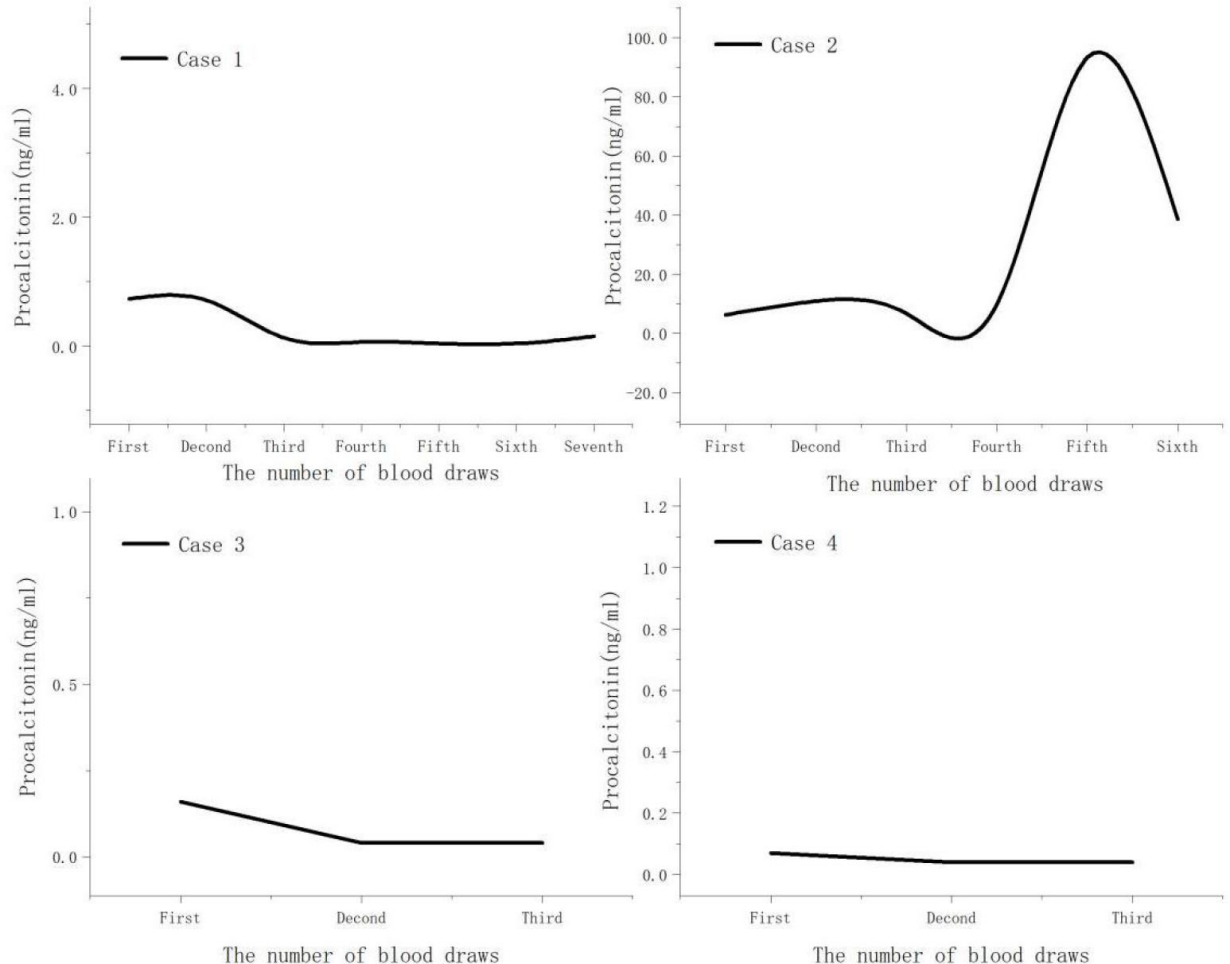
Procalcitonin change curve.

Both case 1 and case 2 were severe cases. On admission, all cases demonstrated significant elevations in C-reactive protein, ferritin, creatine kinase, and lactate dehydrogenase levels (**Table 2**). Additionally, absolute lymphocyte count decreased in all cases, with more clear decreases in case 1 and case 2 (**Figure 1**). Notably, no improvement in absolute lymphocyte count was observed in case 2 (**Figure 2**). Furthermore, the study found that calcitonin levels progressively increased in case 2, indicating a worsening condition. All cases underwent tests, including bronchoalveolar lavage fluid for acid-fast bacteria, fungal G (1,3β-D glucan) and GM (galactomannans) tests, and tumor marker tests, all of which were negative.

**Table 2:** Laboratory findings in patients with Chlamydia psittaci pneumonia.

Main laboratory indicators	Case1	Case2	Case3	Case4
Serum chemistry				
Alanine aminotransferase, U/L	43	55	11	14
Glutamic oxaloacetic transaminase, U/L	54	59	20	16
Lactic dehydrogenase,U/L	811	696	751	246
Creatine kinase, U/L	888	67	414	128
Creatine kinase isoenzyme, ng/ml	11	14	3.53	15
Serum creatinine, μmol/L	40	193	46	69
Blood urea nitrogen, mmol/L	1.6	16.4	<1	4.4
Uric acid, μmol/L	87	308	106	207
Hypersensitive C-reaction protein,mg/L	302.53	272.42	220.05	36.89
Procalcitonin,ng/ml	0.73	6.35	0.16	0.07
Serum ferritin, ng/ml	>1675.6	>1675.6	327.2	427.6
Erythrocyte sedimentation rate,mm/h	72		108	30
Blood routine				
Leukocyte, 10^9^/L	11	12.8	4.2	9
Percentage of neutrophils, %	92.6	96.6	80.4	73.8
Lymphocyte percentage, %	5.5	2.2	14.7	17.3
Absolute value of lymphocytes, 10^9^/L	0.6	0.3	0.6	1.6
Erythrocyte,10^12^/L	3.81	3.13	3.56	5.37
Hemoglobin, g/L	111	98	108	163
Platelet,10^12^/L	207	185	159	200
Blood Gas Analysis				
PH value	7.474	7.434	7.502	
Partial pressure of carbon dioxide mmHg	32.7	30.5	33.7	
Oxygen partial pressure,mmHg	52.1	50.3	91.4	
Hydrogen carbonate concentration,mmol/L	23.8	20.1	26.2	
Base excess,mEq/L	1.1	-2.8	3.3	
Lactic acid,mmol/L	1.3	3.7	0.7	

**Figure 3:**
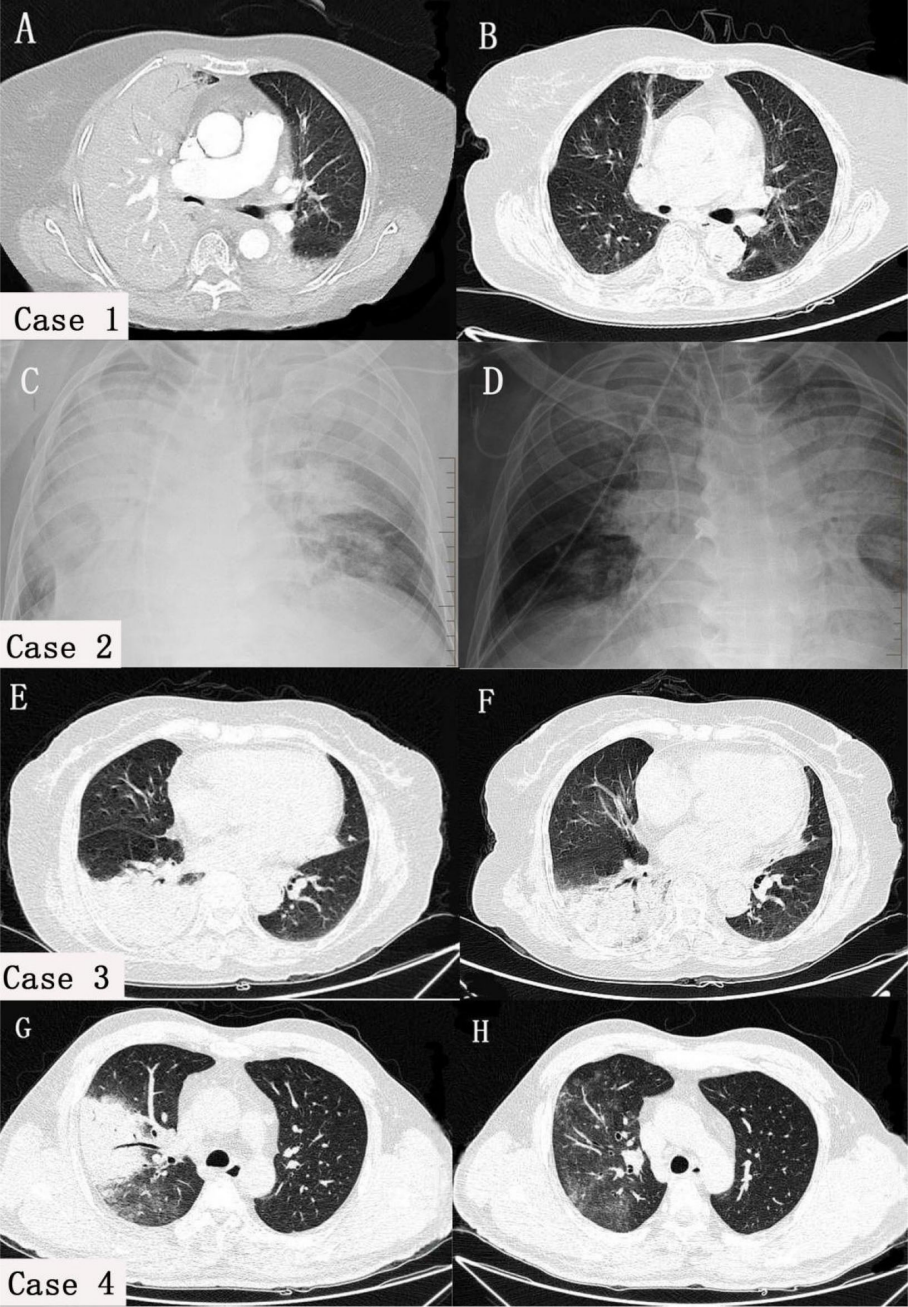
Comparison of chest Imaging scans before and after treatment in four cases. In case 1 **(A)** and case 2 **(C)**, both lungs had pleural effusion, atelectasis, and lesions, with the right lung being mainly affected. In cases 3 **(E)** and case 4 **(G)**, consolidation was predominantly observed in the right lung, with a few flocculent and striate changes in the left lung. After treatment, pleural effusion **(B, D)** and lung consolidation **(F, H)** significantly improved in all cases.

Except for case 2, who underwent bedside chest X-ray examination, all cases underwent chest CT after admission. The results revealed several infectious lesions in both lungs, predominantly lobular consolidation and atelectasis in the right lung, and slight flocculent and striped changes in the left lung. Pleural effusion was observed in case 1 and case 2. Following one week of treatment, pleural effusion markedly decreased in case 1 and case 2. Additionally, the infected lesions were significantly absorbed in case 3 and case 4 (**Figure 3**).

## Pathogenic examination

All cases underwent mNGS testing on alveolar lavage fluid samples obtained through bronchoscopy. mNGS revealed *chlamydia psittaci* infection. Notably, cases 1 and 2 had higher gene sequence numbers than cases 3 and 4. Additionally, cases 1 and 2 underwent blood mNGS testing, which demonstrated a higher number of mNGS sequences in the alveolar lavage fluid compared to peripheral blood (**Table 3**). Phylogenetic analysis of genes in a study on disease control in Lishui City indicated that 11 sequences from four cases were closely related and clustered together on the same branch, indicating high homology (Yao et al., 2022).

**Table 3:** The mNGS results of BALF in patients with Chlamydia psittaci pneumonia.

	Time from onset to test (d)	Type of specimen	pathogen	Number of sequences	Relative abundance(%)	Type of specimen	pathogen	Number of sequences	Relative abundance(%)
Case1	5d	BALF	C.psittaci	7144	77.32	Blood	C.psittaci	71	64.55
Case2	12d	BALF	C. psittaci	7204	78.96	Blood	C.psittaci	6759	78.84
Case3	6d	BALF	C. psittaci	256	79.89				
Case4	4d	BALF	C.psittaci	4418	54.53				

BALF, Bronchoalveolar lavage fluid; C. psittaci, Chlamydia psittaci.

## Treatment and outcome

Case 1 was hospitalized on the third day of onset, while case 2 chose to rest at home and did not seek medical attention. On August 23, case 2 sought medical attention for a pulmonary infection at the local county people’s hospital. There, case 2 received ceftazidime 2.0g ivgtt q8h in combination with moxifloxacin 0.4g ivgtt qd for 5 days. On August 28th, case 2 developed new symptoms such as chest tightness, dyspnea, and difficulty breathing. Blood gas analysis revealed decreased blood oxygen saturation, and follow-up chest CT showed an increased number of pulmonary lesions. Therefore, case 2 was transferred to our hospital for further treatment. Both case 3 and case 4 developed symptoms and were directly hospitalized on the 3rd and 1st days of onset, respectively.

All four cases received combined antibacterial therapy with quinolones before their diagnosis. After being diagnosed with mNGS, levofloxacin was switched to azithromycin for case 1, moxifloxacin was switched to azithromycin for case 2, no adjustments were considered for case 3, and minocycline was added to moxifloxacin for case 4. Following the changes in the treatment plan, the body temperature of all cases returned to normal the day before and after the change. During treatment, case 2 experienced gastrointestinal bleeding, sepsis, and multiple organ failure, which led to sudden cardiac arrest and subsequent death on September 13. On the other hand, case 1, case 3, and case 4 were discharged from the hospital on September 15, September 10, and September 23, respectively.

## Discussion

In 1879, for the first time, J. Ritter reported seven cases of atypical pneumonia caused by Chlamydia psittaci infection (Harris and Williams, 1985). Since then, there have been outbreaks of varying scales in multiple locations. Psittacosis cases are predominantly sporadic, with rare instances of aggregation. Reports of human Psittacosis outbreaks have been documented in European countries, United States, Australia, Japan, and other locations. These outbreaks typically occur in poultry processing factories, bird fairs, and pet bird facilities (Hedberg et al., 1989; Hinton et al., 1993; Lederer and Mx Ller, 1999; Tiong et al., 2007; Berk et al., 2008; Matsui et al., 2008; Belchior et al., 2011; Williams et al., 2013). Reports of familial clustering are extremely rare (Bourke et al., 1989; Kaibu et al., 2006; Çiftçi et al., 2008; Boeck et al., 2016). In 2021, during the COVID-19 pandemic, China first reported a family outbreak of human psittacosis (Li et al., 2021), followed by two additional reports of family clusters experiencing the onset of the disease (Xiao et al., 2022; Jing et al., 2023). These reports all referred to parrots as the source of the disease, and patients were family members who had direct contact with the birds. This article discusses cases who got the infection from a duck. The four cases were not from the same family. One of them was a duck vendor, and the others were a family of three. It is worth noting that two members of the family did not have direct contact with the sick duck. Thus, the transmission of pathogens in this study is more complex and warrants further investigation.

Chlamydia psittaci pneumonia is a zoonosis that primarily affects the lungs but can also damage other organs such as the liver, kidney, circulatory system, and central nervous system, thereby leading to multiple organ failure (Zhang et al., 2020). According to statistics, approximately 1.03% (ranging from 0% to 6.7%) of community-acquired pneumonia is caused by Chlamydia psittaci annually (Hogerwerf et al., 2017). However, in countries such as the United States, the United Kingdom, and the Netherlands, pathogenic microorganisms are not typically tracked in uncomplicated common pneumonia (De Gier et al., 2018). This lack of tracking can lead to misdiagnosis and missed diagnoses of parrot fever pneumonia, and ultimately some patients do not receive effective treatment.

Psittacosis is usually transmitted from birds and poultry to humans, mainly through the respiratory tract or direct exposure to birds’ excretion (Smith et al., 2011). Recent studies have indicated that chlamydia psittacosis can be isolated from mammals such as cattle and horses, which is also infectious (Lenny et al., 2020). Although interpersonal transmission of psittacosis pneumonia is rare, it is still possible. Researchers in China conducted a study on confirmed cases of psittacosis pneumonia and their close contacts in a hospital in Shandong Province. The study revealed that the epidemic originated from birds and was transmitted to humans, followed by second and third-level human-to-human transmission. The transmission also happened through several asymptomatic carriers and medical staff (Zhang et al., 2022). This study reports the first instance of human-to-human transmission of psittacosis pneumonia in China.

In this article, four cases were discussed. One of them was a duck vendor, and the others were family members who bought the duck. The disease manifested in a short period, with case 1 and case 2 starting on the same day. All four cases were confirmed to be infected with Chlamydia psittaci through alveolar lavage fluid mNGS.

Additionally, the disease control team confirmed the homology of the gene sequences of all four cases through nested PCR. The researchers collected 74 samples, including throat swabs, anal swabs, and poultry drinking water. Of these samples, 8 were positive (Yao et al., 2022). Therefore, the diagnosis of psittacosis was confirmed in all four cases. The disease simultaneously manifested in case 1 and case 2 due to contact with diseased ducks. However, the infection transmission in cases 3 and 4 may have been more complex due to various factors: 1) Case 3 and case 4 did not have direct contact with the infected duck; 2) The symptoms of case 3 and case 4 appeared several days after case 1 and case 2 (9 days later for case 3 and 17 days later for case 4). The incubation period of psittacosis is typically 1-2 weeks (Hogerwerf et al., 2020), and the time interval between the infection of cases 1 and 2 and case 4 exceeded the incubation period; 3) Cases 3 and 4 showed the first symptoms when case 2 was hospitalized. Based on the previous reports of person-to-person transmission of psittacosis, it is highly suspected that the infection was transmitted from case 2 to cases 3 and 4 through the respiratory tract. Case 3 and case 4 were also exposed to the home environment, and the disease control unit also confirmed that the environmental samples were positive. In addition, there are reports that the incubation period of psittacosis can be up to 45 days **(** Jenkins et al., 2018). Case 3 and case 4 might have been indirectly exposed to the contaminated environment. Further investigation into the infection pathways of these cases is warranted.

Once inhaled into the lung, Chlamydia psittaci enters the bloodstream and infects the macrophages in the liver and spleen (Fraeyman et al., 2010). From there, it spreads to other organs, manifesting with symptoms such as high-grade fever, chills, headaches, myalgia, and dyspnea. In severe cases, it can quickly progress to atypical or severe pneumonia and even affect other organs (Beeckman and Vanrompay, 2009; Cillóniz et al., 2016). In the absence of timely diagnosis or treatment+, the mortality rate can reach up to 10%-20% (Hogerwerf et al., 2017). In this study, all cases began with high-grade fever, with a peak body temperature of ≥39.5 °C. Cases 1 and 2 had noticeable chest tightness and dyspnea. Acute respiratory failure occurred within 3 days of admission, and tracheal intubation and ventilator-assisted respiration were required. Case 2 ultimately died from cardiac arrest, while the symptoms of cases 3 and 4 were relatively mild. The severe symptoms of cases 1 and 2 were attributed to direct contact with infected ducks. The results of mNGS revealed that case 1 and case 2 had higher mNGS sequences compared to other cases. mNGS was performed for case 2, 12 days after the onset of symptoms, which was the longest interval, but case 2 had the highest sequence number and worst prognosis. The findings suggest that the quantity of mNGS sequences can potentially be utilized to assess the severity and prognosis of diseases. Notably, the numbers of mNGS sequences detected in the bronchoalveolar lavage fluid (BALF) of case 1 and case 2 were higher than that in peripheral blood, indicating that examining BALF via bronchoscopy may enhance the detection rate of pathogens in psittacosis pneumonia.

Metagenomic next-generation sequencing (mNGS) has emerged as a valuable tool in detecting infectious diseases, including psittacosis pneumonia (Expert, 2019). Although its detection rate is significantly higher than traditional methods, mNGS is expensive and not widely used in clinical practice. Typically, it is reserved for cases with undermined diagnosis and severe disease. Studies have shown that tetracyclines, macrolides, and quinolones are effective in treating mild cases of psittacosis. However, in some countries, the diagnosis and treatment guidelines for mild community-acquired pneumonia (CAP) do not require identification of the specific cause of the illness, which can result in delayed treatment of patients with psittacosis. For instance, in the Netherlands, amoxicillin is the conventional treatment for CAP (Postma et al., 2015), but it is not effective against psittacosis pneumonia.

Similar to other atypical pathogens, psittacosis pneumonia has characteristic features on CT imaging, such as localized nodules or patchy consolidation. These lesions progress rapidly to lobar consolidation within 2-5 days and are primarily distributed adjacent to the pleura. Commonly, they are accompanied by pleural effusion and show ‘fine mesh’ signs around them (Chunfang et al., 2023). The ‘anti-halo sign’ can be used as an imaging feature for psittacosis pneumonia (Rongju and Pingchao, 2023). The main findings on CT images were consolidation and atelectasis, and some of the patients had pleural effusion, without obvious “anti-halo sign”.

We observed that CRP and ferritin levels increased to varying degrees in all cases, with the severity of the disease positively correlated with the degree and duration of the increase in these serum markers. This indicates that the pathogen can trigger systemic inflammatory reactions after entering the bloodstream, which may worsen the severity of the disease. Consistent with our findings, previous studies indicated that increased procalcitonin level is significantly associated with severe pneumonia (Simmons et al., 2017). In case 2, the procalcitonin levels progressively increased, ultimately resulting in patient’s death.

Lymphopenia is a significant feature of severe psittacosis pneumonia. It is linked to the immune dysfunction and is usually proportional to the duration of the disease (Yang et al., 2022). In this study, all four cases had decreased absolute lymphocyte count, with cases 1 and 2 exhibiting a significant decrease. Additionally, case 2 showed no improvement in lymphocyte count, indicating severe immune dysfunction. In this study, the lymphocyte count of case 1 began to increase after 20 days of admission, while the lymphocyte count of case 3 and case 4 returned to normal after a week. The severity of the disease was associated with the degree of lymphopenia and recovery time in all patients. Hence, absolute lymphocyte count can serve as a crucial indicator for evaluating disease severity.

In summary, *chlamydia psittaci*, as a common pathogen of CAP, is severely underestimated in clinical practice, and mGNS can become an important means of its detection. Parrot fever pneumonia is mainly transmitted through poultry, but clustered outbreaks are increasing reported. Interpersonal transmission requires high attention and vigilance against the emergence of a human-to-human pandemic. Absolute lymphocyte count and PCT can be important indicators for predicting patients’ prognosis.

## Data availability statement

The original contributions presented in the study are included in the article/supplementary materials. Further inquiries can be directed to the corresponding author.

## Ethics statement

Written informed consent was obtained from the individuals for the publication of any potentially identifiable images or data included in this article. Written informed consent was obtained from the participant/patient(s) for the publication of this case report.

## Author contributions

YW dealt with the case and drafted the manuscript. XX, YL and XJ assisted collected case data and literature and carried out all the documentary and article work out. HW gave some constructive suggestions for this paper during the revision and production period. JY and LH contributed to conception, design, and critically revised the manuscript. All authors contributed to the article and approved the submitted version.
